# Arthroscopic Soft Tissue Procedures for Anterior Shoulder Instability

**DOI:** 10.2174/1874325001711010979

**Published:** 2017-08-31

**Authors:** Mathew Brown, Andrew Wallace, Andrew Lachlan, Susan Alexander

**Affiliations:** Fortius Clinic Central 17 Fitzhardinge Street London W1H 6EQ UK

**Keywords:** Arthroscopy, Shoulder instability, Bankart lesion, Labral reconstruction, Labral repair, Suture anchor

## Abstract

**Background:**

Arthroscopy has rapidly transformed the treatment of anterior shoulder instability over the past 30 years. Development of arthroscopic equipment has permitted the investigation and diagnosis of the unstable shoulder, and fixation methods have evolved to promote arthroscopy from an experimental procedure to one of first-line mainstream treatment.

**Methods:**

Key research papers were reviewed to identify the fundamental principles in patient diagnosis and appropriate selection for arthroscopic treatment. The evolution of arthroscopy is described in this article to facilitate the understanding of current treatment.

**Results:**

Accurate diagnosis of the shoulder instability subtype is essential prior to selection for surgery. Different surgical techniques are described to address different pathology within the glenohumeral joint related to instability and the appropriate method should be selected accordingly to optimise outcome.

**Conclusion:**

Anterior shoulder instability can be treated successfully using arthroscopic surgery, but the surgeon must treat each patient as an individual case and recognise the different subtypes of instability, the associated pathological lesions and the limitations of arthroscopy. The article concludes with a suggested algorithm for the treatment of anterior shoulder instability.

## 1 INTRODUCTION

Arthroscopic soft tissue surgical procedures have become mainstream treatment in the management of anterior shoulder instability. The most important step in any surgical treatment is to establish the correct diagnosis and appropriate patient selection, in order to achieve the best outcome. Instability in the anterior direction accounts for >90% of shoulder instability and may occur as a result of trauma sustained to normal tissues or due to underlying hypermobility that may be secondary to abnormal tissues, and may be further complicated by dysfunction of the neuromuscular structures around the shoulder girdle. It is crucial that the clinician completes a careful history and examination, including observation of posture and effect of the kinetic chain, to establish the underlying cause of instability as this greatly influences treatment.

## 2 CLASSIFICATION OF SHOULDER INSTABILITY

**Fig. (1):**
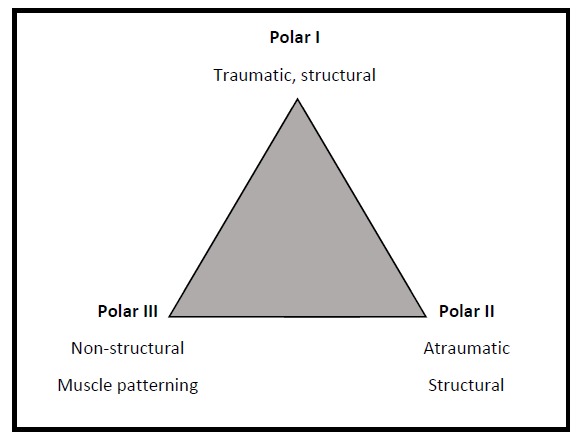
The Stanmore Instability Triangle.

The Stanmore Instability Triangle [1] recognizes 3 subgroups of shoulder instability and allows for the fact that the nature of the instability may change with time. It is important to take this into account when planning surgical treatment to select the appropriate treatment (Fig. **1**).

**Polar I** is the traumatic structural group, which includes patients who have a high energy impact such as a rugby tackle that causes damage to the structures within the shoulder such as labral tear.

**Polar II** is the atraumatic structural group, these patients often present with low... energy dislocations that occur during activities that would not normally result in a dislocation such as throwing a duvet across a bed. These patients also have evidence of some structural damage within the joint.

**Polar III** are those patients who do not have any structural damage within the shoulder and who demonstrate abnormal muscle co...ordination or activation often referred to as ‘muscle...patterning’. These patients are often able to voluntarily dislocate and relocate their shoulders (habitual dislocators).

A patient may initially present with a Polar I injury but with time can develop recurrent episodes of instability that occur with little trauma, so they would then be classified and treated like a Polar II patient. Likewise a Polar III patient may develop structural damage in the shoulder, which may require surgery.

The incidence of traumatic shoulder instability is approximately 1.7% in the general population with increased incidence observed in young men, contact athletes (or those engaged in repetitive over...arm activities), and the military [2]. Hovelius *et al.* reported a 23% incidence of recurrent dislocation (requiring surgical intervention), which increased to 34% in cases aged 12...22 years [3]. Robinson *et al.* reported that young men with Polar I instability had a 55.7% risk of recurrent instability within the first two years and this risk decreased by 82% following surgical repair [4, 5]. Therefore surgical reconstruction is recommended in patients presenting with Polar I instability, however patients presenting with Polar II or Polar III instability should be assessed by a specialist shoulder physiotherapist before embarking on any surgical procedures.

## 3 PATHOANATOMY OF SHOULDER INSTABILITY

Glenohumeral stability relies upon a complex interaction of static and dynamic stabilising factors, including the rotator cuff muscles, capsulolabral structures and negative intra-articular pressure. Damage or dysfunction to these natural constraints may result in recurrent instability, which is associated with pain, disability and a limitation in participation in high level activities [6].

Anatomical structures that provide static shoulder stability include the bony congruency of the concave glenoid and spheroidal humeral head, the fibrocartilaginous glenoid labrum, glenohumeral ligaments, and negative intra-articular pressure [7]. Dynamic stabilisers are primarily muscular and include the rotator cuff (supraspinatus, infraspinatus, subscapularis and teres minor), in addition to the long head of biceps tendon and muscles that stabilise the scapula. The rotator cuff muscles generate a compressive stabilising force that centres the humeral head onto the glenoid, a phenomenon known as ‘concavity compression’ [7]. This mechanism is present in all positions, although particularly important in the functional mid-range when the capsule and ligaments are relaxed.

The glenoid labrum is critical for glenohumeral stability [8] and injury to this structure is identified in almost all cases of recurrent, traumatic anterior shoulder dislocation [9]. The labrum deepens the glenoid cavity, thereby increasing humeral contact area and preventing excess humeral head translation. It also provides an attachment for the glenohumeral ligaments [9, 10].

Ligaments responsible for glenohumeral instability include the superior glenohumeral ligament (SGHL), the middle glenohumeral ligament (MGHL), and inferior glenohumeral ligament (IGHL), Different ligaments are recruited at different stages of the movement arc. The IGHL is the primary restraint to anterior subluxation of the humeral head when the shoulder is abducted to 90 degrees and externally rotated, with the IGHL under tension in this position. The MGHL provides anterior stability in the mid-range of shoulder abduction, whilst the SGHL (in addition to the more robust coracohumeral ligament) resists inferior subluxation of the humeral head when the arm is in neutral abduction, placed by the side of the trunk [11].

## 4 ANATOMICAL LESIONS IN ANTERIOR SHOULDER INSTABILITY

There are a number of anatomical lesions that may occur to the capsulolabral structures as a result of shoulder instability and these are summarised in (Table **1**). [13-15]. Rupture of the subscapularis tendon is a rare cause for recurrent primary anterior glenohumeral instability [16], and occurs more frequently in older patients following anterior dislocation.

A systematic review by Longo *et al.* reviewed 31 studies related to primary anterior shoulder dislocation and identified 19 studies (1,245 shoulders) reporting soft tissue lesions including 415 Bankart lesions (33.3%), capsulolabral detachment in 83 (6.6%) shoulders, 43 SLAP lesions (3.4%), 4 anterior labral periosteal sleeve avulsion (ALPSA) lesions (0.3%) and 2 humeral avulsion glenohumeral ligament (HAGL) lesions (0.2%). Rotator cuff tears occurred in 82 (6.5%) shoulders. Bone defects were reported in 21 of the 31 studies (1,977 shoulders). Defects of the glenoid (bony Bankart lesions) were reported in 35 (1.7%) shoulders (however, Sugaya *et al.* reported a 50% prevalence), whereas defects of the posterior humeral head (Hill Sachs lesions) were reported in 488 (24.6%) shoulders. Fractures of the greater tuberosity were reported in 148 shoulders (7.5%) [17].

Plain radiographs and computed tomography (CT) are useful to assess bony defects. Magnetic resonance (MR) imaging is useful to investigate soft tissue defects in the capsulolabral structures and the rotator cuff muscles [18]. MR... arthrography (MRA) and CT arthrography are especially useful in delineating the type and extent of capsulolabral injuries [19, 20], especially if there has been previous surgery. Results should be interpreted with caution as subtle undetected glenoid damage is recognised as a primary cause of labral stabilisation failure and recurrent instability [21, 22]. Examination under anaesthesia (EUA) combined with diagnostic arthroscopy is the gold standard for diagnosis.

## 5 ARTHROSCOPIC ANTERIOR STABILISATION

The principle of surgical management of symptomatic anterior shoulder instability is to restore soft tissue and bony defects. The rapid technical development of shoulder arthroscopy in the late 1980s led to a greater appreciation of the intra-articular pathology of instability. As visualisation and instrumentation improved, arthroscopy permitted the dynamic visualisation of the entire joint through a full range of movement to assess for damage and recruitment of anatomical structures. Arthroscopic surgery can be performed either under general anaesthesia or regional interscalene nerve block, or both. Patients are placed either in the ‘beach chair’ or the lateral decubitus position. Lateral decubitus positioning requires a longitudinal perpendicular traction device to hold the limb abducted, and therefore there is a risk of iatrogenic brachial plexus injury if excessive traction force is applied. Beach-chair positioning has been linked to cerebrovascular injury, including stroke [23, 24] and visual loss [25], however careful anaesthetic control of blood pressure and cerebral perfusion pressures has improved safety. The beach chair is also preferred if there is an appreciable risk of conversion to open surgery.

## 6 TRANSGLENOID SUTURE REPAIR AND SURETAC

The technique of transglenoid suture repair of the detached anterior labrum was first published by Morgan in 1987, and popularised by Caspari [26-28]. In essence, this technique used a suture passing device to pass multiple absorbable monofilament sutures through the labrum *via* an anterior portal. These sutures were then gathered together and passed through a single trans glenoid drillhole located just below the anterior glenoid margin. A small incision was made posteriorly to tie each suture over the infraspinatus fascia, securing the labrum to the anterior glenoid rim. Whilst initial results were promising, longer-term reports reflected a significant failure rate. Pagnani et al reviewed 41 cases with mean 5.6 years follow-up (range 4-10) and reported 7 cases (19%) of recurrent instability [29]. All failures occurred within the first 2 years. Zaffagnini *et al.* compared arthroscopic transglenoid suture technique and open capsular shift with Bankart repair in 110 cases with minimum 10 years follow-up (range 10-17) and reported no statistical difference between the similarly high recurrence rates (12.5% *vs* 9%) [30]. Variables in tension of tying the sutures over a muscle and fixation through a single point were factors that concerned surgeons adopting this technique.

Around the early 1990s, arthroscopic soft tissue reattachment was made easier by the introduction of absorbable tacks fabricated from polyglycolic acid (PGA, SureTac). With a flanged shaft and spikes located around the periphery of the head of the tack, these devices were cannulated and could be used to fix the labrum to the bone over a guidewire with relative ease [31]. However, these were relatively bulky implants and reports emerged of these tacks loosening, causing impingement, degrading rapidly leading to osteolysis and sterile synovitic reactions due to the hydrolysis of the polymer [32].

Cadaveric, biomechanical and clinical studies showed that following a dislocation episode, not only is the labrum detached but the capsule undergoes plastic deformation, and this aspect also needed to be considered in the design of arthroscopic techniques - *i.e.* it is not just the reattachment and healing of the labrum that is important, but reduction of capsular redundancy is also required to adequately reconstruct the inferior glenohumeral ligament complex [33, 34].

## 7 THERMAL SHRINKAGE

The technique of thermal shrinkage was popularized in the late 1990s as an option for patients with atraumatic (polar type II) instability. It was based upon the application of an alternating radiofrequency current (350kHz to 1 MHz), which results in polarity changes leading to oscillation of water molecules causing friction thereby generating heat. As collagen molecules heat above 55° to 60°C, the intramolecular hydrogen bonds break and the proteins denature resulting in loss of the highly ordered crystalline structure and instead the collagen molecules form random coils [35]. This causes shrinkage in length of tissue, and this concept was used to reduce overall glenohumeral capsular volume and therefore limit movement of the glenohumeral joint and reducing instability. Cadaveric studies were promising and demonstrated an overall 37% reduction of capsular volume and 48% less anterior translation with a 20N load with the joint at 45 degrees of abduction and external rotation [36].

However, the resulting tissue in vivo was biomechanically weaker and replaced by scar tissue, and clinical results were disappointing with Hawkins *et al.* reporting a 43.5% failure rate in 85 cases after a minimum of two year follow up, 26% were in cases of anterior instability plus a Bankart lesion, and 33% (10 of 27) in anterior instability without a Bankart lesion [37]. In another study of 101 consecutive patients treated with thermal shrinkage the direction of instability was associated with failure with 80% failures (4 out of 5) in patients with predominantly posterior instability, 22% (14 of 44) failed in patients with isolated anterior instability and 28% failures in (8 out of 26) multidirectional instability. The overall failure rate was 31% and so this technique has largely been abandoned for shoulder instability [38]. Revisions of thermal shrinkage failures are particularly difficult due to the invariably inadequate residual capsular tissue.

## 8 SUTURE ANCHORS

The next quantum step in arthroscopic management of instability was the development of the suture anchor [39]. A small titanium device fitted with an eyelet carrying a loop of suture was driven into the subchondral bone and secured with single or double flexible metallic arcs, acting like a harpoon or barb to prevent pullout. This concept facilitated multiple points of attachment without the need for long loops of suture tied outside the joint, but necessitated the ability to tie knots inside the joint and around the labrum at each point of fixation.

In 1993, Wolf and others reported improved results with this technique compared to previous published results using tacks or transglenoid sutures [40]. A number of different designs were brought to the market, from screw in to tap - in devices, with varying sizes, pullout strengths and ease of insertion. Titanium is a very biocompatible material, but these devices could cause problems if incorrectly inserted, left proud of the joint surface or were subject to migration, and could be difficult to remove in these situation or if complicated by infection. Nonetheless the introduction of suture anchors allowed the surgeon to both tension the capsule as well as to fix the labrum, effectively reproducing the Bankart repair technique from inside the joint without the risk of open surgery.

Improvements in biomaterials led to the introduction of polymers with longer biological half-lives of degradation and so in the mid-2000s many suture anchors comprised of varying isomers of poly-L-lactic acid (PLLA) were introduced [32]. Although PLLA is a relatively brittle material, once in the bone it degrades over 1-2 years and so provides sufficient initial fixation for labral healing to occur before undergoing absorption. Many papers reporting good results of labral repair with absorbable anchors emerged, using both knotted and knotless fixation methods [4]. In the late 2000s several papers suggested that in some patients (particularly when large numbers of absorbable anchors had been used), the resorption process could lead to osteolysis of the glenoid and possibly contribute to degeneration of the articular cartilage [41, 42] Furthermore in contact athletes with previous absorbable anchor fixation, further injury can be associated with glenoid rim fractures through the regions of osteolysis [43].

More recently anchors composed of poly ether ether ketone (PEEK) have been employed to address some of these issues [44]. PEEK is a strong biocompatible plastic polymer that can be utilised in either screw in or tap in designs, and has been used in medical applications for several decades. The advantage of these implants is that if there are problems with insertion, or complications such as infection, they can easily be drilled out and do not pose major problems in the case of revisions to open surgery if required. However even with PEEK implants there can also be some element of reaction in the adjacent bone leading to occasional loosening or backing out of the anchor.

With the development of composite materials incorporating both degradable polymers and osteoconductive ceramics such as tri-calcium-phosphate [45], many of these issues look to become less of a concern, although longer term studies showing their efficacy in comparison with existing labral repair techniques in humans are lacking. Perhaps the most attractive option is the ‘all... suture’ based anchor, in which the polyethylene suture is deployed on insertion to form a knot which is captured under the subchondral bone plate, eliminating the need for an anchor body to carry the suture [46]. This means the diameter of drillhole can be significantly reduced, and more points of fixation can be achieved without creating additional stress risers in the bone.

Even with the continued technological development of soft tissue re-attachment, these devices still essentially create a single ‘spot weld’ for point fixation of soft tissue to bone. Recently several authors have developed techniques in which the sutures can be linked together to form a suture bridge to improve the contact of soft tissue to bone between each point of attachment. Lafosse initially described a technique (‘Cassiopeia’) of a double row labral repair, although did not publish any clinical results [47]. In a cadaveric study it was shown that this approach more closely reproduced the normal anatomy of the capsulolabral insertion [48]. The development of anchors that facilitate independent tensioning of the sutures, irrespective of the deployment of the anchor in the bone, has allowed a form of chain linkage to tension the sutures between anchors – ‘The Flying Swan Technique’ [49]. This may improve overall strength of the construct, as well as higher contact pressure between bone and labrum and improved footprint of healing. This may be especially useful in ALPSA lesions, where reattachment of the displaced labrum to the bone may result in ‘rim healing’ that may explain the higher failure in reported studies compared to Bankart lesions [50].

## 9 TREATMENT OF BONE LOSS

The pioneering work of Burkhart and De Beer suggested that in patients with significant bone defects of the glenoid or humeral head, the results of arthroscopic stabilisation were poor (up to 67% failure) [51]. Two factors were identified that can be assessed arthroscopically – firstly the presence of an ‘inverted pear’ shape of the inferior glenoid (indicative of erosion or bony Bankart lesion), and secondly the presence of an engaging Hill-Sachs lesion (*i.e.* where the humeral defect slides over the anterior glenoid margin and ‘locks out ‘ or engages preventing spontaneous reduction. This philosophy of designing the appropriate procedure for each individual patient has been expanded to include the concept of the ‘glenoid track’ which is effectively quantification of the overall reduction in joint surface that is due to the combined effect of both the glenoid defect and the Hill-Sachs lesion that results in engagement [52].

From an arthroscopic perspective, two approaches have been proposed to deal with these issues. It has been shown that the open Latarjet technique, either as originally described [53] or as modifed to produce a congruent glenoid arc [54], has an excellent outcome in terms of preventing recurrence, although in systematic reviews has been associated with a 30% overall complication rate [55, 56]. In 2007, Lafosse et al published the first description of arthroscopic Latarjet procedure and reported good short-to mid-term results in 2010 [57, 58].

Boileau *et al.* [59] described an arthroscopic technique of coracoid transfer with encouraging early results, although the operation is long, technically difficult (12% conversion to open) and at present associated with a significant rate of nonunion (17%) and hardware problems. No studies have yet been presented showing any advantage over the open technique, especially in contact/collision athletes.

In dealing with the engaging Hill-Sachs lesion, Purchase and Wolf, devised a technique of ‘remplissage’ to deal with the loss of the humeral articular surface by capsulotenodesis of the posterior capsule and infraspinatus into the defect [60]. This is held in place after debridement of the defect (to promote healing) using one or two anchors. Ideally due the placement of the anchors in the cancellous bone of the humeral head, which is less dense than that of the glenoid, the anchors should be larger in diameter (4-5mm) in order to prevent pull-out. Although theoretically this manoeuvre should cause some limitation of external rotation (by medialisation of the capsule/cuff insertion), that could be an issue in athletes, clinical reports to date have not revealed this to be a problem, with acceptably low recurrence rates of 4-5% [40, 61, 62].

## CONCLUSION

Over the last two decades, arthroscopic methods of soft tissue reconstruction have advanced, partly due to evolution of suture passage techniques, the advent of new biomaterial,s and implant designs, and partly due to improved understanding of the pathomechanics of shoulder instability. The authors currently believe that the evidence base supports the treatment algorithm presented in (Table **2**).

It has emerged, however, that not all lesions can be reliably managed by arthroscopy alone and open procedures still have a role, especially in patients engaged in high-demand or contact sport.

## Figures and Tables

**Table 1:** Anatomical injuries associated with anterior shoulder instability.

**Lesion**	**Description**
Bankart lesion	Avulsion of the IGHL and anteroinferior labrum from the anterior glenoid with a disrupted periosteum.
ALPSA lesion	Anterior labrum periosteal sleeve avulsion is an avulsion of the anteroinferior labrum that is displaced and reflected medially on the glenoid neck.
HAGL lesion	Humeral avulsion of the glenohumeral ligament - peeling off of the IGHL at its humeral insertion.
Perthes lesion	Anteroinferior labral avulsion combined with a peeling off of the intact periosteum from the anterior glenoid neck.
SLAP lesion	Superior labral tear from anterior to posterior, Several subtypes are described including an associated avulsion of the proximal long head of biceps tendon.

**Table 2:** Treatment algorithm for anterior shoulder instability.

Instability Type	Structural Defect	Recommended Surgical Technique
Primary dislocation	normal glenoid bony anatomy Bankart lesion non-engaging Hill-Sachs lesion	arthroscopic anterior labral repair + capsular shift
Primary dislocation	normal glenoid anatomy Bankart or ALPSA lesion engaging Hill-Sachs lesion	arthroscopic labral repair (Flying Swan technique) + remplissage
Recurrent dislocation	normal glenoid anatomy, Bankart or ALPSA lesion, non-engaging Hill-Sachs lesion	arthroscopic labral repair (Flying Swan technique) +/- remplissage
Recurrent dislocation	any glenoid bone defect, Bankart or ALPSA lesion, engaging Hill-Sachs lesion	initial scope then proceed to open modified Latarjet technique

## LIST OF ABBREVIATIONS

AMBRIA: = Traumatic multidirectional, bilateral, rehabilitation, and occasionally requiring an inferior capsular shift
CT: = Computed tomography MRI Magnetic resonance imaging
HAGL: = Humeral avulsion of the glenohumeral ligament ALPSA Anterior labrum periosteal sleeve avulsion MDI Multidirectional instability
MRA: = Magnetic resonance arthrography EUA Examination under anaesthesia ISIS Instability severity index score
SGHL: = Superior glenohumeral ligament MGHL Middle glenohumeral ligament IGHL Inferior glenohumeral ligament
SLAP: = Superior labral avulsion from anterior to posterior
TUBS: = Traumatic unidirectional, Bankart, and usually requiring surgery
WOSI: = Western Ontario Shoulder Instability Index
